# Do patients and research subjects have a right to receive their genomic raw data? An ethical and legal analysis

**DOI:** 10.1186/s12910-020-0446-y

**Published:** 2020-01-16

**Authors:** Christoph Schickhardt, Henrike Fleischer, Eva C. Winkler

**Affiliations:** 1 National Center for Tumor Diseases (NCT), Department of Medical Oncology, Heidelberg University Hospital, Heidelberg, Germany; 2 Institute for German, European and International Medical Law, Public Health Law and Bioethics (IMGB), Universities of Heidelberg and Mannheim, Mannheim, Germany

**Keywords:** Genomic, Raw data, Release, Access, Right, Ethics, Law, Recommendations, Informational self-determination, General data protection regulation (GDPR), Privacy

## Abstract

**Background:**

As Next Generation Sequencing technologies are increasingly implemented in biomedical research and (translational) care, the number of study participants and patients who ask for release of their genomic raw data is set to increase. This raises the question whether research participants and patients have a legal and moral right to receive their genomic raw data and, if so, how this right should be implemented into practice.

**Methods:**

In a first step we clarify some central concepts such as “raw data”; in a second step we sketch the international legal framework. The third step provides an extensive ethical analysis which comprehends two parts: an evaluation of whether there is a prima facie moral right to receive one’s raw data, and a contextualization and discussion of the right in light of potentially conflicting interests and rights of the data subject herself and third parties; in a last fourth step we emphasize the main practical consequences of the ethical analyses and propose recommendations for the release of raw data.

**Results:**

In several legislations like the new European General Data Protection Regulation, patients do in principle have the right to receive their raw data. However, the procedural implementation of this right and whether it involves genetic counselling is at the discretion of the Member States. Even more questions remain with respect to the research context. The ethical analysis suggests that patients and research subjects have a moral right to receive their genomic raw data and addresses aspects which are also of relevance for the legal discussion such as the costs of release of raw data and its impact on academic freedom.

**Conclusion:**

Taking into account the specific nature and implications of genomic raw data and the contexts of research and health care, several concerns and potentially conflicting interests of the data subjects themselves and involved researchers, physicians, biomedical institutions and relatives arise. Instead of using them to argue in favor of restrictions of the data subjects’ legal and moral right to genomic raw data, the concerns should be addressed through provision of information and other measures. To this end, we propose relevant recommendations.

## Background

As Next Generation Sequencing (NGS) technologies are increasingly implemented in biomedical research and (translational) care, the number of study participants and patients who ask for release of their genomic raw data is set to increase. Yet, despite an intense discourse on the (potential) ethical, legal and social implications of the progressive application of NGS, little attention has been paid to the release of genomic raw data. Research participants express interest in receiving raw data from participation in NGS studies [1]. Wright et al. report that the UK 100,000 Genomes Project Protocol warrants patients the right to request that their whole genome sequencing data be made available to them [2]. Some general tendencies might contribute to a growing demand for personal access to raw data: i) Use of NGS platforms continues to expand. ii) Younger generations are more confident with the use of IT and handling of data. iii) Whereas the return of single potentially health relevant findings (incidental or additional findings) is dependent on researchers making and reporting back such findings with patients and participants playing a passive role, copies of raw data can be *actively* requested by patients and participants. iv) The growing number of private companies offering genomic data analysis on a commercial basis as well as of non-profit initiatives, for instance data sharing initiatives [3, 4], might also encourage requests for one’s personal raw data.

The authors of the few publications and comments on the release of raw data express a wide range of positions: Bredenoord et al. [5] consider the release of raw data to participants to be “non-sensical”. Some authors reject the release of raw data to individuals unless several conditions such as, for instance, specific and appropriate resources from funders, are met [1, 2]. Others recognize the right but suggest that it might be denied by Access Offices or Research Ethics Committees [6] or strongly support it [7–9]. Some suggest that it might be a valid alternative to reporting back incidental or additional findings of individual health relevance making the report of such findings to individuals potentially unnecessary [1, 9].

Before beginning the analysis, some preliminary clarifications are necessary. By the term “genomic raw data” we refer to large sets of genomic data generated by High Throughput Sequencing of the whole genome or parts of the genome, for instance the exome. We rely on two (interconnected) criteria to characterize the term: first, the data must display the status of “rawness” in the sense that the data must stem from a rather initial state of production or analysis. Presuming a scale of increasing elaboration and scientific analyses of data from their initial production by NGS core facilities to the final interpretation of their scientific or individual meaning, raw data stem from the first steps of this scale. Second and *ex negativo*, raw data are clearly distinct from specifically interpreted or annotated data such as findings of individual health relevance. It is not possible to define a comprehensive list of raw data formats. As Thorogood et al. [6] put it, “data types and formats may differ depending on the context, sequencing platform, analysis pipeline, and evolution of common file formats”. Examples of genomic raw data sets include the data generated by a Next Generation Sequencer such as FASTQ files, Binary Alignment/Map format (BAM files) or Variant Call Format files (VCF files), i.e. variant bases relative to the reference genome [2]. In our understanding, the term raw data thus also comprehends sets of the so-called differential genome: the set of all not further specified variants between the personal germline genome or exome and the reference genome or exome, or the set of all not further specified variants between a patient’s tumor genome or exome and the patient’s germline genome or exome. Finally, “rawness” of data should not be naively understood in the sense of “natural” or “neutral” with respect to scientific and technological methods, conditions and assumptions: raw data too are a cultural (i.e. social, scientific, technological) construction and no neutral or objective reflection or representation of “nature as it is” [10].

NGS platforms have traditionally been implemented in biomedical research but are increasingly finding application in translational and clinical contexts. To deal with our question, distinguishing between the research and care contexts is therefore necessary even though we are aware of the notorious difficulties associated with the research-treatment distinction and the fact that many applications of NGS techniques like NGS based translational research projects or NGS diagnostic studies occur somewhere in between. We schematically understand one pole of the scale as “pure research”, i.e. research activities with the sole goal of gaining new generalizable scientific insights, and the other pole as “pure clinical care”, i.e. diagnostic and treatment activities pursuing the sole goal of understanding and improving the individual health conditions of a patient [11]. The general normative distinction between research and care is closely related (even though not necessarily congruent) to another relevant distinction: whereas NGS applications in clinical care require clinical validity and approval, research uses of NGS do not [12].

### The international legal framework

As to international treaties, the Convention on Human Rights and Biomedicine of the Council of Europe (the ‘1997 Convention’) [13] contains important principles on the rights of affected individuals across the field of biomedicine. Pursuant to Article 10 (2) of the 1997 Convention, *‘everyone is entitled to know any information collected about his or her health (…)*’. The Additional Protocol to the Convention on Human Rights and Biomedicine concerning Genetic Testing for Health Purposes states that ‘*everyone undergoing a genetic test is entitled to know any information collected about his or her health derived from this test’*. Furthermore, Article 13 (2v) of the Protocol on Biomedical Research mentions the need for ‘*arrangements for access to information relevant to the participant arising from the research and to its overall results*’ and ‘*any other personal information’* (cf Article 26 (2)). However, it is not evident, whether the right to know guaranteed by these regulations relates only to “information” in the sense of de-facto knowledge, as opposed to raw data, from which information and knowledge would, after all, still need to be extracted. As very few states have ratified these instruments it may be more useful to turn to the binding laws of the European Union.^1^ For brevity’s sake, U.S. legislation cannot be examined in detail here, meaning that this paper focuses on the laws of the European Union. However, remarkably, the significance of the recently implemented General Data Protection Regulation (the ‘GDPR’) extends beyond European borders, especially with regard to data transfers to countries outside the European Economic Area (‘third countries’). In this context, the determining factor is whether the level of data protection in the third country is adequate, in other words whether it is comparable to that of the GDPR.

Pursuant to the decisions of the European Court of Human Rights (the ‘ECHR’), Article 8 of the European Convention on Human Rights (the ‘Convention on Human Rights’) (‘*Right to respect for private and family life’*) contains not just obligations, such as guaranteed access to medical records [14], but also the positive right to obtain copies of one‘s medical record [15]. The Convention on Human Rights and the decisions of the ECHR serve as an interpretative aid to the member states when it comes to determining the ambit and extent of fundamental rights and fundamental legal principles at the level of their own constitutional law [16]. Article 8 of the Convention on Human Rights also forms a base of Article 8 of the European Union’s Charter of Fundamental Rights (protection of personal data) (the ‘CFREU’). Under Article 8 (2) CFREU, *‘everyone has the right of access to data which has been collected concerning him or her (…).’*

These value judgments, made by European Union law, influence the above­mentioned GDPR, which succeeds the Data Protection Directive. Accordingly, Article 15 GDPR provides for a right of access by the data subject, in order to be aware of, and verify, the lawfulness of any processing of their data. Raw data is clearly included within the ambit of Article 15 (3), as this addresses the data controller’s duty to provide a copy of the personal data undergoing processing (although the controller may charge a reasonable fee based on administrative costs for any additional copies requested by the data subject). Furthermore, Article 20 GDPR grants a right to data portability. This means that the data subject will have the right to receive their personal data in a structured, commonly used, and machine-readable format.

Recital 63 of the GDPR can be used as an interpretative aid in this context: It is in line with the ECHR’s decisions, as it states that a data subject’s right of access includes their right to access health data, such as the data contained in their medical records including diagnoses, test results, doctor’s assessments and evaluations, and any treatment given or interventions made. This would suggest that raw data, which were generated in a biomedical context, are intended to be included in the definition, given that such data contains information relevant to genetic disorders and dispositions. Notably, this would not be limited to treatment, but include data collected in a research context.

However, Article 23 (1 i) GDPR allows restrictions to be placed on this right under EU law or the laws of the Member State to which the data controller or processor is subject, as long as *‘the restriction respects the essence of the fundamental rights and freedoms and is a necessary and proportionate measure in a democratic society to safeguard the protection of the data subject or the rights and freedoms of others’*. This is most likely intended to refer to cases where there is a therapeutic necessity to do so. For example, Member States would have the power to provide that raw data could only be released by way of genetic counselling [11]. Further grounds for placing a restriction on the right of access are given by the freedom of research, which is a fundamental right guaranteed under article 13 of the CFREU. Art. 89 (2) GDPR provides that EU or domestic law may provide for derogations from the right of access where personal data are processed for scientific or historical research purposes, in so far as such rights are likely to render impossible or seriously impair the achievement of the specific purposes, and to the extent that such derogations are necessary for the fulfilment of those purposes. Member States are therefore able to pass laws and regulations which permit research institutions to refuse the release of raw data to the relevant data subject in situations where doing so would mean a serious risk to, or prevention of, a research objective. Art. 15 (4) GDPR, which states that the right to obtain a copy of one’s data under Article 15 (3) shall not adversely affect the rights and freedoms of others, can also be used as a basis for restrictions in favour of scientific research. However, interestingly it would not restrict the right of patients to receive their raw data on the basis of the potential rights of their genetic relatives. If this were the case, it would be impossible to afford individuals the right to genetic testing in the first place as genetic testing bears an even greater risk of impairing the right not to know or the right of informational self-determination of the genetic relatives of the patient tested.

To conclude, due to the GDPR patients of sequencing studies do in principle have the right to receive their raw data. However, the procedural implementation of this right and whether it involves genetic counselling is at the discretion of the Member States. It is provided that in exceptional circumstances, the right can be restricted in order to balance the right of data access with the freedom of research. For this reason, the application of the right to the research context, i.e. beyond medical care, is less evident, as Member States can provide corresponding derogations here that require a weighing of interests in each individual case.

The release of genomic raw data is a new challenge for law and praxis. Beyond the Law of the European Union, Thorogood et al. [6] report that in the US the question whether persons have a legal right to access their genomic raw data in contexts of biomedical research with the raw data not being stored in the health record is not completely clarified. In many countries, the legal right to access genomic raw data in the research sector is object of more restrictions and/or often unclear. As to the UK, Kaye et al. stated in 2013 that “participants in genomic research under current UK law have very limited rights to access” their personal genomic data. International and national research ethics guidelines are widely silent about access to genomic raw data in research [6, 17]. So in the field of law several questions with respect to the right to access and its interpretation and implementation remain open. Beyond its independent value in its own right, the following ethical analysis can also inform the discussions of these questions and relevant developments in the law.

## Methods

### The moral prima facie right to genomic raw data

We begin the ethical analysis with some general reflections on the nature of privacy which will lead us to assert a prima facie right to receive one’s genomic raw data. The ethical analysis will be carried out from the perspective of rights-based egalitarian liberalism. The moral basis of liberalism is equal respect for persons [18]. This basic principle is designed to treat persons as beings capable of autonomously thinking and acting based upon reasons [18] and translates into a primacy of liberty rights [19, 20]. The burden of justification is with those who restrict liberties [19]. Liberalism responds to the fact that reasonable people disagree on the idea of the good. It emphasizes respect and toleration to secure everybody the exercise of personal and political freedom [21]. *Egalitarian* liberalism responds to the fact that people need substantial means and capacities to appreciate and exercise formally warranted liberties as it suits best for them [20].^2^

As genomic raw data are personal, sensitive and inherently identifying, the question of a right to receive one’s raw data is to be subsumed under the right to informational self-determination or the right to privacy. The value of privacy can be identified in an intrinsic value or in the function of privacy for other rights and values, that is in an instrumental (extrinsic) value [22, 23]. Our approach is based upon an instrumental understanding of privacy as playing a key role for protecting and fostering liberal rights and values of importance for single individuals and society: personal autonomy and self-determination [23–25], inviolate personality [26] and dignity, human well-being and individual flourishing [27, 28], social relations [29], equality and democracy [30–32].^3^Informational privacy or informational self-determination certainly constitutes a core element of privacy [23, 33, 34]. The right to informational self-determination protects a person’s ability to freely decide whether and how personal data and information about her are collected, stored, multiplied, processed and transferred by third parties [25, 27, 32, 33]. In the following, we use the term informational self-determination instead of (informational) privacy for it better captures that the right is about *actively* determining what happens with one’s personal data and information.^4^

To effectively exercise the right to informational self-determination, the data subject depends on several single elements, for instance the right to give or reject consent to the collection of personal data by third parties. The right to receive a copy of data stored by others is one element of the right to informational self-determination. First and in accordance with the reasoning of the EU-GDPR the right is necessary in order to know what data other people have about me – this is again a condition for an informed exercise of the right to informational self-determination. Second, the data are data about that person [34], containing personal information about the person (the body, physical and mental dispositions) and information relevant for the person’s self-knowledge, individual lifestyle and life planning. Personal data like genomic data bear an inherent and non-alienable persisting relation to the data subject. Third, the right avoids an undesirable informational asymmetry given when third parties have personal data of potential relevance for the person’s self-understanding, personal narrative, identity or autonomy, that the person herself lacks. Such an informational asymmetry is contrary to the liberal ideal of equality and emancipation and might also be a potential source of non-transparent and non-legitimized power, influence or manipulation. From these general considerations we conclude that data subjects have a prima facie right to receive a copy of their genomic raw data.

One criticism might object that the argumentation so far does not take into consideration the nature of genomic data nor the context. Responding to this objection gives us the opportunity to clarify our approach in general and in particular with respect to the role of context. We have only argued that there is a prima facie right to access genomic raw data. For an adequate ethical analysis of the matter, taking into account the context related aspects – the implications of genomic data, the rights, interests and roles of persons involved – is indispensable and will be addressed in the next section. However, even the limited normative weight we attribute to the prima facie right might be challenged by competing approaches to privacy and the role of context within these approaches. As to the ethical discourses on privacy and informational self-determination, Helen Nissenbaum’s privacy-as-contextual-integrity-approach [33] can be considered the most prominent one that attributes a particular importance to the context and criticizes too general accounts of privacy.

Nissenbaum’s privacy-as-contextual-integrity-account [33] is based upon two central tenets: first, it assumes that every arena or realm of social life is governed by implied and well-established social norms of information flow: “there are no arenas of life *not* governed by *norms of information flow*, no information or spheres of life for which ‘anything goes’.” Second, it claims that these social norms together with the social practice and context they occur in, offer a normative account: “any of these sources [distinct types of contexts, domains, spheres institutions, or fields] could provide a foundational concept for articulating the concept of contextual integrity in relation to personal information”. Existing informational norms are morally binding and should be respected [33]. Confronted with Nissenbaum’s approach, we specify ours as follows: i) we do not start from a one-principle or a one-value-account of privacy, but from a more comprehensive normative framework (liberal egalitarianism) and a more complex account of privacy within this framework: privacy as an instrumental value within the system of liberal values and rights; ii) we *will* take into consideration the context and context-related aspects; iii) however, we are more cautious and skeptical with respect to the normativity of contextual informational rules, for the following two reasons: First, we think that existing informational norms internal to certain social practices are mainly ethically demanding from an *ex-post* perspective: When one engages in a certain kind of social practice one explicitly or implicitly communicates to the other persons involved that one agrees and commits to implied existing informational rules governing that practice. The ethical reason demanding respect to the implied informational rules after having engaged in a particular social practice such as, for instance, friendship, are not specific or limited to information flows, but are based on the moral duties of honesty, reliability, trustworthiness and respect to concluded agreements. Similar to a judge’s perspective on court litigations, the contextual integrity approach appears to fit best for evaluating cases of the past. Second, the strategy of attributing normative demandingness to existing social rules as such might lead to a too conservative approach (“presumption in favor of the status quo”). It lacks normative potential for ethically motivated revisions of existing social practices as well as for regulations of relatively new social phenomena, such as, for instance, requests for genomic raw data. In contrast, we address the release of raw data in certain contexts and social relations according to what these contexts and social relations ideally *should* be like and with a view to formulate policy recommendations for the future handling of release of raw data.

## Discussion

### The moral prima facie right in context

Due to specific aspects of genomic raw data and the contexts of treatment and research, there might be reasons to question the data subject’s prima facie right to receive genomic raw data. Such reasons arise from concerns regarding the interests of the data subjects themselves and potentially conflicting rights and interests of other persons and parties involved: the researchers, physicians, the biomedical institutions and family members.

### The data subject

Concerns and objections building upon the interests of the data subjects themselves claim that receiving raw data is not useful or that it is even potentially harmful for data subjects. Studies suggest that people have a great interest in accessing their raw data [1, 35, 36]. However, the largest relevant study also shows shortcomings in people’s understanding of genomic raw data: among the (non-professional) public who were keen to receive personal raw data, many said that, if they were given their genomic raw data, they would analyze the data themselves or ask their general practitioner or primary care physician for analyses. As both the lay public as well as general practitioners are unable to analyze and interpret genomic raw data, this raises the concern that people “may have perceived genome sequence data to be both simpler and more interpretable than it actually is” [1].

Data subjects’ potentially non-adequate understanding of the nature of genomic raw data plays a key role in concerns and objections that have been put forward to emphasize the uselessness or potential harm of raw data for the data subject [1, 2]. This way of reasoning can also be referred to as “Knowledge-deficit argument”. As to the utility, it is clear that in contrast to single findings with annotations concerning health relevance, raw data as such do not constitute information ready to be transformed into (medical) actions. Retrieving information of clear health relevance for the individual from genomic raw data requires sophisticated bioinformatic tools and highly specialized competencies in human genetics. Almost all people, even health professionals, lack such tools and competencies. Additionally, at least to date, the sequencing data produced in research or translational contexts are of limited reliability for they are not necessarily produced with clinically validated and approved methods. Beyond its questionable utility, the release of raw data might even be a source of psychological, social or economic burdens and harms for the individual data subject. Data subjects might be disappointed when they learn that the data are not what they erroneously took them to be, for instance that they are not easily interpretable genetic information ready for use. The data subject’s handling of the raw data might undermine the confidentiality and security of the data. This could enable other persons to (legally or illegally) gain sensitive personal information and result in ideal or material harms if the information is used against the data subject, for example by (future) employers, insurance companies, marketing and publicity companies or state agencies. Furthermore, genetic findings such as those that can be retrieved from raw data are often probabilistic, associated with uncertain clinical diagnosis and treatment options (actionability) and thus complicated to understand and communicate. These difficulties hold also for the communication of genetic information to relatives, which is another potential source of burdens for the data subject who, when passing genetic risk information to relatives, must also take into account the relatives’ right not to know about genetic dispositions.^5^

As to the empirical basis of these concerns of lacking utility and potential harm, it is worth pointing out that there exist no representative studies on the understanding of genomic raw data and personal experiences of persons who really requested and received their raw data [2]. References to the poor understanding of many lay persons also neglect that the understanding of people can change; indeed, we will argue that (poor) understanding of the nature of raw data should be addressed and improved by targeted information during the release process. Also, to deal with their lack of tools and competencies in interpreting their own raw data, data subjects can refer to professional bioinformaticians and geneticists from the public health and research sector, to patient driven organisations or to private companies offering analysis and interpretation of genomic data as a service. Public and commercial services for genome interpretation are not all and necessarily of poor quality, or, as Angrist [8] puts it: “to pretend that CLIA-certified testing and analysis are still the only game in town seems a bit naïve this late in day.”^6^ Beyond medical utility (for health, clinical care or reproduction), genomic data may also serve personal and social interests of the data subject, for example as entertainment, learning, way to relate to others or as option for purely potential usage in the future [1, 36].

Assessing the concerns and objections referring to data subjects themselves on a more theoretical and normative level, we first want to emphasize that most references to potential lack of utility and to risks of burdens and harms for data subjects are not based upon the perceptions and values of the data subjects, but on what others presume to be good or bad for the data subjects. Determining what is good or bad for others without knowing their opinions, values and life plans (and taking decisions for others on this basis) is sometimes necessary, for instance with respect to very young children. Yet, from a liberal perspective, every person is considered to know best what is good or bad for her and should have the right and responsibility to decide for herself what kind of conception of a good life and plans to follow [38]. To repudiate or restrict a person’s liberties on the basis of a determination of what is good or bad for her without relying on the person’s opinions and will is ethically problematic and challenging to justify. To restrict or deny a person’s self-determination in order to protect her from (self-induced) harm qualifies as what is usually referred to as paternalism. In some cases, paternalistic interventions may be justified. However, to be justified, paternalistic interventions must meet several conditions to a sufficient degree: the person to be treated paternalistically must be badly informed; the potential harm to be avoided through the paternalistic intervention must be relatively likely to occur and of substantial magnitude, i.e. threatening the person’s life or important resources and capacities to plainly use her liberties in the future; there must be no other reasonable alternative option to avoid the harm or protect the person without violating her self-determination and liberties. This criticism (lack of respect for persons’ individuality and liberties, and unjustified paternalism) also holds for the knowledge-deficit argument which – without information about the data subject’s personal values, plans and goals – starts with the presumption that the data subject’s understanding of the nature of genomic raw data is not adequate. Finally, implementing a general rule that denies the liberty of all in order to protect just some persons is additionally problematic and challenging to justify. Not recognising the right of data subjects to access their raw data would prevent even those subjects who could handle that data without (unconsciously) inducing risks or harms to themselves. Overall, the conditions to justify paternalistically motivated violations of data subjects’ prima facie right to raw data are not met.

One might also argue that, still, instead of releasing genomic raw data, biomedical institutions should offer the return of individual genetic information based upon a list of serious and actionable genetic conditions (such as the list elaborated by the American College of Medical Geneticists [39]. However, *as long as* offering the return of individual genetic findings is understood as an *exclusive* alternative which *justifies not releasing genomic raw data* to subjects, we dismiss it for the following reasons: First, as other before mentioned objections it relies on an “objective” determination of the “real” relevance and “real” utility of genomic raw data for people (defined as individual information based upon a determined list of clinically actionable genetic variations). For many persons, release of raw data and return of results will not be equivalent alternatives, or, as Lunshof et al. [7] put it:” There is a crucial difference between providing access to data and returning results”. Second, we take the release of raw data as a moral *right* of persons that cannot be cancelled or circumvented by the bound party through “offering” some other (and remarkably different) thing – not to mention the specific challenges, responsibilities and costs in terms of genetic counseling and other resources that biomedical institutions would incur in case of a return of results policy. Third, at least in EU member states, persons do have, in terms of principle, a *legal* right to receive their genomic raw data.

We conclude this section by stressing that, rather than addressing the concerns regarding data subjects’ lack of understanding, limited utility and potential harms from raw data release by means of prohibitions, restrictions or forced “alternatives”, they should be addressed through offering general elementary information to help data subjects make an informed and autonomous use of their right which suits their personal interests and way of life. That is why we advocate that the release of raw data be accompanied by (non-obligatory) information offers – which must not be confused with individual genetic counseling.^7^

### Researchers, research institutions and research participants

We will now scrutinize concerns and objections to data subjects’ prima facie right to receive raw data which rely on (potentially) conflicting rights of other persons and parties involved, starting with researchers and research institutions. So far, we have argued that data subjects have a prima facie right to receive their raw data in a way which allows them to make an informed use of the data. Accordingly, this means that researchers conducting genomic research have the correlative duty to release the raw data to participants in a way to help them to understand the general elementary nature and implications of raw data and their request. Additional reasons in favor of research participants’ right to raw data can be retrieved from a closer look at the ideal of the researcher-participant-relation. The researcher-participant-relation should be understood as a cooperative relation between two citizens and partners who interact in the spirit of equality and reciprocal emancipation and respect.^8^ By this we do not mean that there are no de facto differences, asymmetries and inequalities between the researcher and the participant, for instance with regard to competencies to understand genomic data. We rather claim that the two should interact with due respect for each one’s rights and equality and feel bound by the ideal of partnership and civil fraternity.^9^

Against their duty to release raw data in an appropriate manner, researchers might claim that the pertaining costs (in terms of financial resources and work load etc.) keep them from doing their job and duty, i.e. carrying out research [1], and that it jeopardizes the success of their research projects. Potential costs are the main reason for Wright et al. [2] when they “conclude that unless the return of genomic sequence data to individual participants is specifically and appropriately resourced, potentially be research funders themselves, to do so could do more harm than good”. Addressing the costs concern, Lunshof et al. [7] argue that the costs for raw data release are limited. However, they do not take into consideration that the release should meet certain criteria and thus requires more resources. We understand the process of adequate handling of requests and release of raw data to roughly encompass the following work load: 1. technical part: identification of the data subject’s raw data, production of a copy of the data, quality control, annotation of the copy in terms of data format and quality, making the raw data available to the data subject; 2. Coordination: authentication of the requesting data subject [6], maybe de-pseudonymization of the genomic data set; record of communication with the data subject; 3. Communication and information work: passing general basic information to the data subject, personal discussion with the data subject on demand. Overall costs (including around 50€ for the storage medium for the copy) might amount to around 170€.^10^ Needed resources in terms of time might be higher for the first requests but are likely to decrease with increasing routine and increasing availability of tools [6], models and recommendations for best practice. As NGS biomedical research projects are cost intensive, it appears possible but highly unlikely that such relatively low costs could jeopardize the success of the research project – even if a relatively high number of participants request their raw data.

In cases in which costs are unsustainable for the research project, charging participants with a fair part of the costs is an acceptable solution. Yet, when charging participants, the costs should not be prohibitive, not exclude economically disadvantaged populations [5] and be calculated solely on the basis of the additional costs associated *with the release* of the raw data, not on basis of the costs caused by the production of the raw data initially carried out for the sole sake of research. It has been argued in favor of the release of raw data that it serves the interests of researchers and the scientific community by means of fostering trust and public involvement [34] and through producing a “tangible return of investment in the form of participants goodwill and support for research” [8]. However, these points appear to be rather speculations on possible consequences and might hold, if holding at all, rather for the system of research as a whole than for single researchers or research projects.

Researchers could also object that the release of “their” data violates their academic freedom. However, first, as mentioned above, the data are not theirs; second, it is not plausible that the release of raw data could - through costs or other consequences - prevent or discourage scientists from engaging in what is at the core of academic freedom: to freely choose research questions and methods, carry out research and publish results without being hindered, intimidated or discriminated against. Academic freedom does not ground a monopoly and property right on collected personal data from data subjects [41]. Third, in research on human subjects, academic freedom needs to be balanced against the rights of those subjects. When compared to other and well-recognized elements of the right to informational self-determination within biomedical research such as the right to withdraw consent to research usage (including the right that the data be deleted), the right to receive a copy of one’s raw data represents a minor disturbance for research.

Another concern refers to unfair competition and market distortion. If a publicly sponsored biomedical institution releases raw data to data subjects free of charge (or just charging the costs of the release of the genomic raw data as described above), this might negatively affect private for-profit companies which offer the sequencing and releasing of one’s genome as a commercial service to customers. However, to date, this does not seem very realistic, one reason being that private companies such as *23andMe* do not confine their service on solely sequencing the genome, but offer genetic analysis and interpretations that are explicitly not part of the release of raw data as understood in this article. However, the worry of market distortion and unfair competition might be a reason why publicly sponsored genomic research projects should renounce to recruit research participants through advertising the possibility of release of raw data free of charge. Likewise, charging research participants some of the costs associated with the release of raw data could be a way to partially respond to the concern.

Contrary to Middleton et al. [1] who suggest that the release of raw data might be an alternative option to reporting back individual health relevant findings, including clinically actionable results, we strongly advocate that releasing raw data shall not abrogate researchers’ ethical duty to report back incidental or additional findings of significant health relevance to the individual participant.^11^Understanding it as alternative option appears to assume that every finding that a highly specialized researcher might make during a cutting edge research project (and with maybe additional information about the participant) could be equally discovered and interpreted by the participant herself through referring to available services or tools. The position seems to assume also that every participant requiring and obtaining raw data has the economic and intellectual capacities to refer to genetic testing services, which warrant high validity and quality of the data and interpretation, and actually refers to them (within time). These assumptions are hardly realistic. Respect to the right to raw data is no matter of good will or generosity, but a moral duty which cannot be offset against respect to other duties of different content.

Researchers and research institutions have a vital interest to protect themselves in advance against potential and unjustified allegations by the participant or third parties in case of negative consequences from the participants’ handling of their raw data. To prevent harms to reputation and clarify ethical and legal liabilities and responsibilities we suggest that releasing research institutions may and should require participants to sign a very short acknowledgment (“receipt”; see section III). To conclude this section, researchers’ rights and interests do not justify restrictions or derogations of participants’ right to raw data except for the requirement to sign the short acknowledgment (“receipt”).

### Physicians and patients

Looking at the context of medical care we need to examine patients’ right to raw data within the physician-patient relationship and how patients’ requests might concern physicians’ role and rights. Patients (with exception of extraordinary, mainly psychiatric, cases; see the legal section) have a widely recognized ethical and, as seen in the legal section, legal right to access *all* data and information generated and collected during their treatment – to which we refer here as “medical data”. From an ethical viewpoint, patients disclose medical data within the patient-physician relation, which is a relation of asymmetric power, competencies and dependencies. The logic and goal of the physician-patient relation is that of medical care and does not include nor ground physicians’ property or monopoly rights regarding the patients’ medical data. Treating patients as subject and partner^12^ and respecting patients’ autonomy entails the duty to meet patients’ request of access to their data.

One might nonetheless argue that patients’ general right to access all medical data does not apply to genomic raw data. For instance, one could argue that to date genomic raw data are not stored in the electronic patient record or in the clinical information system. However, this argument is too technical and misses the sense of the patient’s right to access all personal data and information collected or produced during the course of care. One might also object that within the medical context genomic raw data should be handled in an exceptional way - as individual genetic findings should be. In Germany for instance the Genetic Diagnostics Act (§ 7) requires that genetic findings are passed to patients only by genetically trained physicians through genetic counseling. In line with this reasoning one might argue that genomic raw data must not be released to patients unless through a process of individual genetic counseling. To this we respond that, as shown above, there are essential differences between genomic raw data on the one hand and single findings of individual health relevance on the other. Single findings might be retrieved from genomic raw data, but this requires further analyses and working steps (by third persons) and does not occur necessarily. Middleton et al. [1] refer to the physicians’ duty to give patients the information they want or need *in a way they can understand* it. From this duty they conclude that physicians and clinician researchers are not allowed to issue raw data to participants of clinical studies for this would entail giving information to patients that they cannot understand (due to the quantity and complexity of the data). Yet, this way of interpreting and referring to physicians’ duty to appropriately inform patients is misleading. The duty’s overall goal is to strengthen patients’ autonomy and rights, not to serve as a justification to restrict them. The duty applies to information deemed relevant for the diagnosis and treatment and to be discussed with patients, not to *all* medical data collected and generated during the course of care.

### The data subject’s relatives

A data subject’s genomic data also concern the data subject’s relatives. Close relatives share roughly 50% of the genome, so that genomic raw data can be used to identify relatives or to learn (with a 50% likelihood) about their genetic dispositions. A data subject’s genomic data thus concern the relatives’ right to informational self-determination. Furthermore, if through further analysis and interpretation of the raw data the data subject learns about a hereditary genetic disposition and passes this information inappropriately over to relatives, this can lead to a violation of the relatives’ right not to know about genetic risks and to social and psychological burdens and harms. However, relatives might also benefit from appropriately being given genetic information by the data subject about serious but actionable genetic dispositions. Even more than in the case of the concerns regarding the data subject herself, potential negative consequences for relatives’ rights are rather hypothetical for they do not stem from the release itself but from *potential* actions (or omissions) by the data subject and other persons subsequent to the release of raw data. To conclude, the implications for relatives do not justify not releasing raw data to data subjects; however, the implications bear sufficient ethical weight to require awareness building efforts by means of being addressed within the information and individual discussion offered to data subjects requiring their raw data.

### Recommendations

The main application oriented ethical consequences from the above analysis are:

- data subjects (research participants and patients) have a right to receive their genomic raw data;
- the right must be respected in a substantial way that helps data subjects to make an informed use of their right and released data;
- concerns relating to the data subjects themselves, researchers, physicians and relatives should be addressed through an information process and do not justify a refusal to release genomic raw data.

Hence, we propose the following recommendations for the practice of release (with most recommendations intended to legally and ethically shape the *upcoming* practice of handling requests for genomic raw data right from the beginning, and some recommendations urging for immediate changes of the *current* practice of planning and funding NGS research projects):

1. basic information on general and elementary features and implications of genomic raw data – to be clearly distinct from individual genetic counseling – should be offered to the data subject as follows: first, when data subjects request genomic raw data, they should be briefly informed about the data’s sensitivity for themselves and their relatives and the complicated usability and limited reliability; second, if data subjects continue to wish to access the data, they should be offered written information (addressing again these points and further aspects) as well as personal discussion with a professional.^13^ Accepting the information and discussion offer is not obligatory for data subjects in order to receive their raw data;
2. the receiving data subjects should be required to sign a very brief document (“receipt”) in which they declare that they were given the data on their request and after being offered basic information about the general nature and implications of raw data and that they assume responsibility for the consequences that might come from their handling of the raw data for themselves and third parties;
3. the raw data files to be released should have a common format that allows for further processing and be accompanied by meta-information about quality and methods used in acquiring it [2].

To prepare for raw data requests in the future:

4) future NGS research projects should consider in advance the possibility of participants requiring their raw data when planning grant submissions and projects, and set up a work flow;
5) the right to receive genomic raw data should be mentioned in the informed consent and be clearly distinguished from plans for the return of individual findings [6];
6) funders should provide funding for potential costs of appropriate release of raw data.

## Conclusion

Requests by patients and research participants to receive their genomic raw data are increasing and can be reasonably expected to continue to increase in frequency in the future. Under some legislations, patients have a general right to access their health data, but several questions remain. From an ethical viewpoint, persons have a moral prima facie right to receive a copy of their genomic raw data which holds also when we take into account concerns and potentially conflicting interests of the data subjects themselves and involved researchers, physicians, biomedical institutions and relatives. Instead of using them to argue in favor of restrictions of the data subjects’ right to raw data, the concerns should be addressed by means of providing information to data subjects to foster their understanding, autonomy and awareness concerning potential implications for themselves and others. To this end, we propose recommendations.

## Data Availability

Not applicable; this article is not based on any collection, analysis or interpretation of data or other empirical material.

## Abbreviations

CFR’s: Charter of Fundamental Rights
ECHR: European Court of Human Rights
EU: European Union
GDPR: General Data Protection Regulation
IT: Information Technology
NGS: Next Generation Sequencing
PhD: Doctor of Philosophy (academic degree)

## Footnotes

1 Due to lack of space and to the mainly ethical (and not legal) nature of the paper, we do not aim to offer an analysis of the law applicable to the question of release of raw data in single EU member states. Analysing the applicable law in single EU member states would need to meet the challenge of interpreting national law and its relation to the new GDPR (for this challenge with respect to German national law, and for the applicable law in Germany see [11]). To our knowledge there is no national law which specifically and explicitly addresses the release of genomic raw data. However, the national biobanking legislation of Finland, enacted in 2013, specifically addresses the management of genetic/genomic data and encompasses a right of access which appears to apply to the release of genomic raw data as well [12].

2 Here, we cannot offer an account of the foundations of egalitarian liberalism nor discuss its (presumed) advantages in relation to other ethical-normative approaches. We therefore confine our explications to two exemplary differences of the account with regard to the so called principlism approach by Beauchamp and Childress: first, in cases of conflicting values, egalitarian liberalism provides a clear priority, i.e. liberty, whereas the principlism approach has notorious difficulties in systematically balancing or assigning priorities. Second, due to its originating from legal and political philosophy, (egalitarian) liberalism is a right-based approach and thus allows for a clear attribution of moral rights and duties which in turn is helpful for the elaboration of application-oriented recommendations.

3 This list of values, in which privacy has been referred to as having a functional value, is far from being a comprehensive reconstruction of the literature on privacy. The entanglement of privacy within liberal rights and values occurs on a conceptual level, when privacy is considered to be implied in the concept of other liberal rights and values as well as on a factual level as an empirical condition which enables or fosters values such as personal autonomy or democracy.

4 In the article, we use both terms “data” and “information”. We tend to use the term “information” as referring to something (a datum) which was interpreted and bears a clear meaning or relevance for somebody. This meaning attributed to the term “information” is illustrated by formulations such as “information relevant to genetic disorders and dispositions can be retrieved from genomic data”. However, we found it hard to strictly differentiate between data and information and to maintain the distinction throughout the text, one reason being that the EU-GDPR does not draw a clear difference between the terms and that normative concepts such as “informational self-determination” use the term “information” or “informational” even though they need to be understood as referring to data as well.

5 See [9]; potential burdens and harms of the release of raw data are mentioned for instance by [1, 34, 37] and are to some extent similar to intensively discussed potential burdens arising from the return of individual findings.

6 Of course, private and commercial services for genetic analysis raise other legal, ethical and social concerns.

7 Even though the information offer encompasses written information and personal discussion as informed consent procedures do, its normative function is clearly distinct from informed consent. Whereas informed consent is required to justify the intervention into a person’s right, the information offer we advocate shall help the data subject to make a possibly well-informed use of her right to receive raw data. Below we will add that the information offer shall also raise the data subject’s awareness of potential implications of raw data for the data subject’s relatives. Of course, the difference concerning the normative function do not warrant that problems of understanding notoriously linked to informed consent processes do not also occur within the information offer we advocate for the process of releasing genomic raw data. This remains a challenge even though we might presuppose that a data subject actively requiring release of raw data might display a higher degree of personal motivation with respect to understanding things than patients and participants in usual informed consent situations do.

8 See [7–9] and, more in general, [40].

9 For fraternity as additional principle for equality among citizens, see [20].

10 It appears plausible that the technical work, the coordination and the communication/information work require half an hour of labour force each, so that all together they might require around 1,5 h amounting to a total of around 120€ for labour force (calculating 6000€ per months for a professional in terms of costs for the employer, one working hour amounts to 37,50 € [6000€ for 160 working hours per month] for the employer). Coordination and communication should preferably be carried out by the same person, for example a project manager with a background (and PhD) in Medicine, Genomics or Molecular Biology. The data might also just be made accessible through an identifier via internet [35].

11 For the claim that researchers have a moral duty to report back incidental or additional findings of substantial individual health relevance and associated with clinical actionability, see for instance [42, 43].

12 For physician-patient-relation as partnership see [44, 45].

13 For detailed recommendations see [11].
